# Creation of Gold Nanoparticles in ZnO by Ion Implantation–DFT and Experimental Studies

**DOI:** 10.3390/nano10122392

**Published:** 2020-11-30

**Authors:** Jakub Cajzl, Karla Jeníčková, Pavla Nekvindová, Alena Michalcová, Martin Veselý, Anna Macková, Petr Malinský, Adéla Jágerová, Romana Mikšová, Shavkat Akhmadaliev

**Affiliations:** 1 Department of Inorganic Chemistry, University of Chemistry and Technology, 166 28 Prague, Czech Republic; jenickok@vscht.cz (K.J.); nekvindp@vscht.cz (P.N.); 2 Department of Metals and Corrosion Engineering, University of Chemistry and Technology, 166 28 Prague, Czech Republic; michalca@vscht.cz; 3 Department of Organic Technology, University of Chemistry and Technology, 166 28 Prague, Czech Republic; veselyr@vscht.cz; 4 Nuclear Physics Institute of the Czech Academy of Sciences, 250 68 Řež, Czech Republic; mackova@ujf.cas.cz (A.M.); malinsky@ujf.cas.cz (P.M.); jagerova@ujf.cas.cz (A.J.); miksova@ujf.cas.cz (R.M.); 5 Department of Physics, Faculty of Science, J.E. Purkinje University, 400 96 Ústí nad Labem, Czech Republic; 6 Institute of Ion Beam Physics and Materials Research, Helmholtz Zentrum Dresden-Rossendorf, 01328 Dresden, Germany; c.akhmadaliev@hzdr.de

**Keywords:** gold, ZnO, nanoparticles, ion implantation, luminescence, DFT, RBS

## Abstract

Three different crystallographic orientations of the wurtzite ZnO structure (labeled as *c*-plane, *a*-plane and *m*-plane) were implanted with Au^+^ ions using various energies and fluences to form gold nanoparticles (GNPs). The ion implantation process was followed by annealing at 600 °C in an oxygen atmosphere to decrease the number of unwanted defects and improve luminescence properties. With regard to our previous publications, the paper provides a summary of theoretical and experimental results, i.e., both DFT and FLUX simulations, as well as experimental results from TEM, HRTEM, RBS, RBS/C, Raman spectroscopy and photoluminescence. From the results, it follows that in the ZnO structure, implanted gold atoms are located in random interstitial positions —experimentally, the amount of interstitial gold atoms increased with increasing ion implantation fluence. During ion implantation and subsequent annealing, the metal clusters and nanoparticles with sizes from 2 to 20 nm were formed. The crystal structure of the resulting gold was not cubic (confirmed by diffraction patterns), but it had a hexagonal close-packed (*hcp*) arrangement. The ion implantation of gold leads to the creation of Zn and O interstitial defects and extended defects with distinct character in various crystallographic cuts of ZnO, where significant O-sublattice disordering occurred in *m*-plane ZnO.

## 1. Introduction

Gold nanoparticles (GNPs) and their special properties have recently attracted considerable attention, especially because of their potential application in antibacterial coatings thanks to their biocidal action [1,2,3]. Their various forms, in combination with different matrices, promote an alteration in the physical, chemical and biological functionalities of these nanoparticles as well as the matrices [4]. For example, such properties as photoluminescence, electrical conductivity, chemical and biological reactivity of the host matrix change as a function of the content, size and shape of GNPs [5]. The remarkable optical properties of GNPs are connected with the plasmonic concept related to the enormous near- and far-field enhancement of the collective excitations of free electrons in gold. Separated as well as embedded nanoparticles have recently been used for many applications in biosensing, intracellular gene regulation, photonics and catalysis [6,7,8]. Solid gold is usually the most stable as a face-centered cubic (*fcc*) structure. In some cases, it has been shown that the growth of nanoobjects (2D layers) on a suitable substrate such as graphene or germanium can change the gold structure to the hexagonal close-packed (*hcp*) arrangement [9,10,11,12]. Since Au(*hcp*) structural arrangement is rare, there are no studies on its properties or the properties of Au(*hcp*):ZnO composites.

Nevertheless, ion implantation offers an alternative approach for the fabrication of GNPs with excellent physical and chemical stability in various solid targets, including dielectric materials and nonlinear crystals. The method, in combination with GNPs, enables the localization, enhancement, and manipulation of electromagnetic fields and charge transfers of substrates, resulting in a range of unique optical phenomena [13]. Benefiting from these advantages, numerous studies have applied ion implantation to fabricate noble-metal nanocomposites for nonlinear plasmonic devices with waveguiding ability [14]. Another advantage of this method is the easy control of the concentration-depth distribution of the Au within the selectable energy and fluence of the implantation. Post-implantation annealing, in combination with the properties of the target structure, is crucial for radiation-damage recovery and subsequently for the functionality of the synthesized composite layer. The target-structure recovery, as well as the structure and position of the implanted ions, are also affected by annealing conditions such as temperature and annealing atmosphere [15].

Gold nanoparticles were first successfully synthesized in the hexagonal wurtzite zinc-oxide (ZnO) structure as a wide-bandgap semiconductor characterized by an optical-bandgap width of 3.4 eV by the electrodeposition technique [16]. Apart from ion implantation [17,18], many other growth methods have been used to prepare the Au:ZnO composite as well. Examples of successful syntheses include chemical deposition from solution, magnetron sputtering, laser ablation, sol–gel coating and thermal annealing, and RF sputtering. In comparison with them, ion implantation offers a certain advantage in the precise localization of gold nanoparticles in mono- and nanocrystalline ZnO structures, and it is now used for the doping of ZnO nanostructures. The possibility to combine such excellent properties as the antimicrobial properties of ZnO and the plasmonic response of noble-metal nanoparticles enables the development of optical sensing systems for the fast detection of biomolecules while providing an antifouling surface, ensuring that the biosensor does not lose its sensing ability over time [19,20]. However, it is clear that during the ion implantation of Au into ZnO, the two materials will interact with each other. It has been reported that the change in the optical properties of ZnO is complex and not yet accurately described. On the contrary, it can be assumed (according to our previous results) that in the rigid hexagonal structure of ZnO, which does not allow easy incorporation of ions, the structure of gold nanoparticles could also be affected.

Regarding theoretical DFT (density functional theory) simulations of Au-doped ZnO, the number of publications is very limited. One of the older works was dealing with the oxidation states of gold atoms on the ZnO surface (which is used for its catalytic action in industrial use) to investigate how gold atoms in a variety of oxidation states may be stabilized on the ZnO surface when substituting Zn^2+^ in the surface layer [21]. Other papers deal with gold clusters on ZnO surface [22] and gold incorporation in ZnO nanowires [23] and ZnO mesocrystals [24,25]. Overall, the results indicate that even a small amount of Au (some Au atoms replacing Zn atoms) produces a strong effect on the electronic properties of the ZnO nanowires (NWs). One of the more recent studies of Au-doping of ZnO investigated two main positions of Au in the ZnO – substitutional site of Zn and interstitial site of the octahedral void [26]. From the results, it follows that according to Au formation energies the Au atoms will more likely take interstitial sites rather than Zn sites. The bandgap was found to decrease for both Au positions in ZnO.

Theoretical DFT modeling of systems similar to our theoretical study has evolved in the past decades, and interesting new approaches emerged. One of the promising ways to calculate defects embedded in various crystals is a ”synthetic growth concept“ (SGC) in which a certain larger portion of the crystal (cluster, i.e., a supercell method), with a dopant(s) embedded in certain positions together with known defects, is by means of iterative geometry optimization process allowed to relax and the final cohesive energies are calculated. By comparison of cohesive energies, a suitable structural arrangement can be iteratively found. Such an approach was applied, e.g., to fullerene-like systems with embedded carbon, sulfur and phosphorus atoms [27,28].

This paper provides a theoretical and experimental study of the location and structure of gold implanted in the wurtzite structure of ZnO in different crystallographic planes. It summarizes and complements our previous experimental results [29,30,31] with DFT (as well as FLUX – Monte Carlo simulation code [32]) theoretical study. It monitors the influence of the experimental conditions of ion implantation and subsequent annealing on the formation, shape and distribution of nanoparticles as well as on the structure of gold nanoparticles. It also analyses the structural changes and damage caused by the impact of Au ions on the differently oriented planes of the ZnO single crystal and the impact of these changes on the optical properties of ZnO.

## 2. Theoretical Procedures

The structural properties of Au-doped ZnO were investigated, and the most favorable positions of Au in the crystal structure were identified using a theoretical structural modeling approach based on a density functional theory *ab-initio* calculation method with generalized gradient approximation (GGA) with Vanderbilt-type ultrasoft pseudopotentials. Theoretical calculations focused both on the energetics of simulated Au-doped crystal models and on the geometrical arrangements in the vicinity of Au ions. The calculations of the energies of the structures (i.e., cohesive energies and Au defect-formation energies) were used for the identification of the most favorable Au positions in the crystals studied. Geometry optimization calculations were mainly utilized for the investigation of the optimal geometrical arrangements in the vicinity of Au ions. The presented theoretical calculations functioned as a basic theoretical approximation of the studied Au-doped crystalline materials; they did not provide a rigorous theoretical study that would account for different aspects of the incorporation of impurities in the crystalline materials (e.g., various numbers of vacancies, different charge-compensation models, etc.). The procedure was the same as in our previous study involving erbium as a dopant for ZnO [33].

The Cambridge Serial Total Energy Package (CASTEP) [34] was used to perform structure geometry optimizations and to obtain the accurate values of the total energies of the respective supercell structural models. The results were obtained as static (0 K), and atomic positions were relaxed according to the calculated forces on each atom. The lattice parameters and atomic positions of crystals were set as the experimentally reported values [35]. In order to perform the calculations with the highest precision, it was necessary to choose a correct exchange and correlation functional (XC) for the given ZnO structure type. The PW91 (Perdew-Wang 91) [36] XC functional was selected for the simulation of Au-doped ZnO from our previously made comparison of different exchange-correlation (XC) functionals based on calculated cohesive energies of tabulated data for undoped ZnO [33].

Rutherford backscattering spectroscopy in channeling mode (RBS/C) is an analytical method utilized for the characterization of crystalline disorder; the probing of He^+^ ions can also serve for the investigation of the channeling effect, which is influenced by the modification of the crystalline matrix. The FLUX code was applied for that. It is a Monte Carlo simulation code [32] that computes the trajectories of the He^+^ ions moving through the crystal during RBS/C measurement. FLUX enables the simulation of 2D maps of ions penetrating through the channels as well as of backscattering ion 2D maps in which a modified crystalline (disordered) structure can be, to some extent, considered. The structural modification of the single-crystal matrix influences the channeling effect of He^+^ ions during RBS/C analysis, where angular scan modification as the narrowing, normalized yield enhancement appears as a consequence of a certain type of defect which caused enhancement of the de-channeling yield can appear [16,37]. The 2D maps of backscattered 2 MeV He^+^ ions were along <0001>, <11–20>, and <10–10> axes in ZnO with implemented Zn-sublattice disorder, affecting the He-ion channeling. The simulations were performed for a perfect ZnO lattice and for a lattice with a relative amount of disorder obtained experimentally from RBS/C and implemented into the ZnO lattice as vacancies at the appropriate implanted depth, conforming to the defected layer identified by RBS/C. The appropriate simulation parameters can be selected based on the nature of the structural modification. In the case of the prevalent nuclear stopping, the vacancy creation seems to be the most employed mechanism of modification. On the other hand, for electronic stopping, the vacancy creation is suppressed, unlike the internal stress induced in the host matrix, which can be imitated by the vibrational amplitude modification [38].

## 3. Experimental Procedures

### 3.1. Ion Implantation

The ZnO single-crystals wafers (10 × 10 × 0.3 mm^3^) with <0001>, <10–10> and <11–20> crystallographic orientations were purchased from CRYSTAL, Berlin, Germany. The samples with the surface along the crystallographic planes (0001), (10–10) and (11–20) are labeled as *c*-, *m*- and *a*-plane, respectively. Au^+^ ions accelerated to 0.4 MeV, 1 MeV or 5 MeV were implanted into the ZnO substrates. The ion-implantation fluences used for the mentioned single-crystal ZnO were 5 × 10^14^, 1 × 10^15^ and 1.5 × 10^16^ ions/cm^2^. Implantation experiments were carried out at room temperature at the incoming ion-beam angle of 7° (off-axis geometry) at the Helmholtz-Zentrum, Dresden-Rossendorf, Germany (the Au-ion energy of 0.4 MeV at the ion implanter) and at the Nuclear Physics Institute of the Czech Academy of Sciences, Řež, Czech Republic (the Au-ion energies of 1.0 and 5.0 MeV at the Tandetron accelerator).

The implanted samples were subsequently annealed in O_2_ at 600 °C for 1 h. The temperature of 600 °C is suggested to be sufficient to avoid the strong vacancy clustering observable at lower annealing temperatures and to reduce the low out-diffusion of a soluble dopant at the temperatures close to 1000 °C [33]. Simultaneously, ZnO has a tendency to lose oxygen from the surface and decompose, the effect of which can be observed well below 1000 °C in damaged ZnO after the implantation [39]; oxygen atmosphere has thus been chosen to protect the surface during the annealing.

### 3.2. Samples Characterization

Using a beam of 2 MeV He^+^ ions from a Tandetron accelerator, the Au concentration-depth profiles, as well as the disorder-depth profiles that occurred during the ion implantation and subsequent annealing, were examined by Rutherford backscattering spectrometry (RBS) and RBS/channeling (RBS/C) measurements, respectively. The signal of the impurity and host lattice in RBS spectra is separated by scattering kinematics. An Ultra-Ortec PIPS detector (Meerbusch, Germany) recorded He^+^ ions scattered at a laboratory scattering angle of 170°. A random spectrum was recorded using a defined rotation of the single-crystalline sample mounted on a two-axis goniometer allowing the precise rotation with the resolution of 0.01°. From such a random spectrum, it is possible to evaluate the gold concentration-depth profiles. The angular scans, modifying the incoming He^+^-ion-beam angles and following the normalized yields of the matrix element Zn and dopant Au, were performed to follow the ion-channeling effect in the prepared samples with various crystallographic orientations of ZnO as well as to study the preferential positioning of Au in the ZnO matrix.

Samples for transmission electron microscopy (TEM) measurement were prepared by the following procedure. The surface layer from the implanted side of the sample was scratched by a diamond indenter, and the removed particles were ground by the Agate pestle and mortar. The powder was mixed with isopropanol (Penta, Prague, Czech Republic) and dropped on the lacey carbon 300 mesh copper grid (Agar Scientific, Essex, UK). After the sample was dried, the Au nanoparticles were extracted by applying 7 µL (one drop) of 5 wt % aqueous solution of citric acid (Penta, Prague, Czech Republic) on the grid. After 40 min, the solution was removed by absorption on cellulose wadding. The grid was washed three times by distilled water and twice by isopropanol and allowed to dry. The samples were observed by Jeol 2200FS (Freising, Germany) field emission electron microscope equipped with an in-column energy filter with a TVIPS camera and EM-Menu software and with an Oxford Instruments EDS analyzer. The contrast of Au nanoparticles in the EDS analysis was enhanced by a high annular angular, dark field (HAADF) detector in the scanning mode (STEM) with a step size of 1 nm.

Raman analysis was performed on a DXR Raman microscope spectrometer (Thermo Fisher Scientific, Waltham, MA, USA) of the company Thermo Scientific equipped with a confocal Olympus microscope. A solid-state Nd:YAG laser (wavelength of 532 nm) was used for the Raman spectroscopy measurement. The measurement conditions were the power of 9 mW, five accumulations of 5 s scans, grating with 900 lines/mm and an aperture with a 50 µm slit. A multichannel thermoelectrically cooled CCD camera was used as a detector. 50× magnification provided a measurement spot-size of ~1 µm^2^. Three measurements were performed on each sample, with the resulting Raman spectra being averaged.

Photoluminescence measurements were performed using a setup that was based on a HORIBA Jobin Yvon Fluorolog^®^-3 Extreme spectrometer (HORIBA France SAS, Longjumeau, France) using FluorEssence™ 3 software. The spectra were collected at room temperature within the range of 250–1000 nm for visible and near-infrared regions (VIS-NIR). Luminescence detection was performed using a photomultiplier tube (PMT) with thermoelectric cooling and a Ce:InGaAs photocathode. The samples were excited by a 450 W xenon continuous-wave (CW) lamp. For the selection of various wavelengths, a double-diffraction-grating monochromator was used at the entrance and a single-diffraction-grating monochromator at the exit. Photoluminescence spectra were collected in a reflection arrangement, with the samples being tilted at an angle of approx. 60°. For spectral evaluation, all of the measured luminescence spectra were transformed to the base level and, after the subtraction of the background, they were normalized.

## 4. Results

### 4.1. The Theoretical DFT Modeling of Au Positions in the Wurtzite ZnO Structure

Since calculations with primitive undoped crystal cells were performed in our previous study [33], the values have been transferred here. Single-point energy calculations (as well as geometry optimization calculations) with Au-doped crystals were carried out with supercell models using the PW91 XC functional selected based on a comparison of cohesive energies. The following parameters were used in the calculations of Au-doped ZnO: the plane-wave cutoff energy of 380 eV, k-point separation in the Brillouin zone of 0.07 Å^−1^ (which for 2 × 2 × 2 supercells corresponded to one irreducible k-point Γ), 300% empty bands (as compared to the valence bands), the smearing of 1.0 eV, the self-consistent field (SCF) tolerance of 5 × 10^−7^ eV/atom. The geometry optimization convergence criteria were the following: the energy change of 5 × 10^−6^ eV/atom, the maximum force of 1 × 10^−2^ eV/Å, the maximum stress of 2 × 10^−2^ GPa and the maximum atom displacement of 5 × 10^−4^ Å.

**Figure 1:**
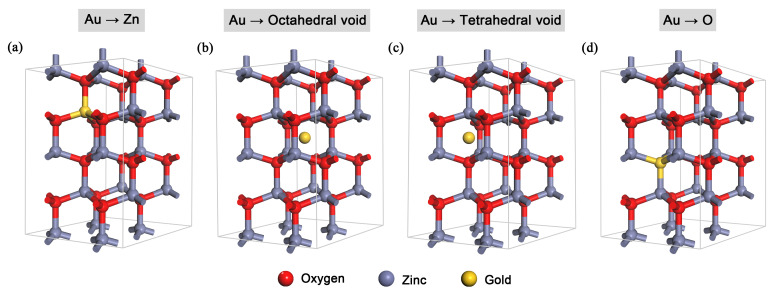
The structural models of Au:ZnO with the gold ion in (**a**) the substitutional position of zinc (Au–Zn), (**b**) the interstitial position of the octahedral void (Au–OctVoid), (**c**) the interstitial position of the tetrahedral void (Au–TetrVoid), (**d**) the substitutional position of oxygen (Au–O). The structural models are 2 × 2 × 2 supercells.

DFT simulations were performed with the ZnO lattice parameters and the atomic positions set to the experimentally reported values: a = b = 3.2494 ± 0.0002 Å, c = 5.2054 ± 0.0002 Å, α = β = 90°, γ = 120°, and space group No. 186 (P6_3_mc) [35]. In the zinc oxide structure, it is possible to encounter three main sites for the gold atom—the substitutional position of zinc and the interstitial positions of octahedral and tetrahedral voids—plus one less probable location, namely the substitutional position of oxygen (see Figure 1), because ZnO, unlike other oxides, also has oxygen vacancies. The fractional coordinates of atoms in the ZnO model, together with four different Au positions in ZnO, are summarized in Table 1.

Geometry optimization calculations were performed with 2 × 2 × 2 supercell models (based on a non-primitive ZnO cubic unit cell consisting of 32 atoms), involving one atom of gold per the total number of 32 or 33 atoms for the respective models. The gold concentration in the structural models was 3.125 and 3.030 at % for substitutional and interstitial models, respectively.

In order to compare different structures in terms of their stability, cohesive energy was chosen as a key quantity. The cohesive energies and defect-formation energies were calculated according to Equations (1) and (2) stated in our previous study [33] with Er-doped ZnO.

The calculated cohesive energies of the Au:ZnO structural models together with the calculated defect formation energies are summarized in Table 2, where Au–Zn and Au–O are the gold atoms in the substitutional positions of zinc and oxygen, respectively, and Au-OctVoid and Au-TetrVoid are the gold atoms in the octahedral and tetrahedral interstitial positions, respectively. The values of cohesive energies and gold defect-formation energies were calculated both before and after geometry optimization.

**Figure 2:**
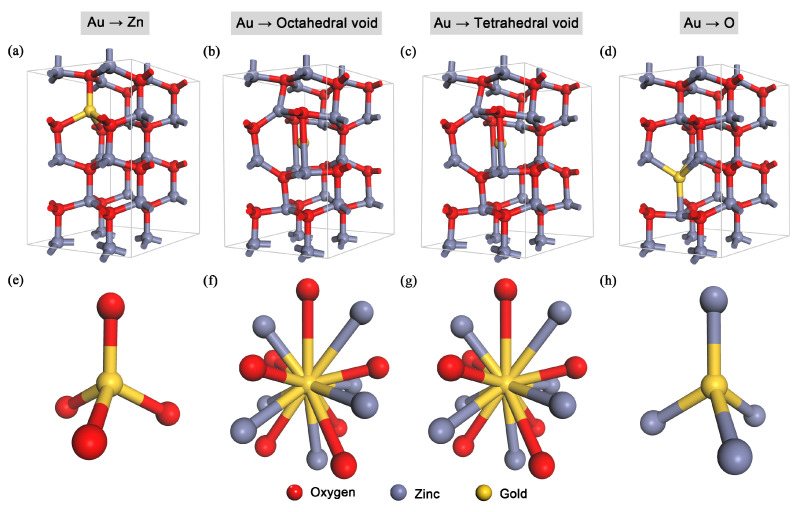
(**a**–**d**) The 2 × 2 × 2-supercell structural models of Au:ZnO with the gold ion in (**a**) the substitutional position of zinc, (**b**) the interstitial position of the octahedral void, (**c**) the interstitial position of the tetrahedral void, (**d**) the substitutional position of oxygen; (**e**–**h**) the vicinity and coordination of Au after the geometry optimization for the respective Au positions in the ZnO structure.

The geometry optimizations (see Figure 2) showed that Au in the substitutional positions of Zn and O formed tetrahedral arrangements, while the interstitial positions of octahedral and tetrahedral voids formed the same complex high-coordination-number structural arrangements with gold. The model with Au in the substitutional position of zinc had the gold coordination number IV, i.e., distorted tetrahedral geometry, with Au connecting to four neighboring oxygen atoms.

The calculations indicate that the models with gold in the substitutional position of zinc (Au-Zn) as well as in both interstitial (octahedral and tetrahedral) positions (Au-OctVoid and Au-TetrVoid) are more likely to form because of higher cohesive energies and lower gold defect-formation energies than the model with gold in the substitutional position of oxygen (Au–O). This finding was expected because oxygen in ZnO has a negative oxidation state while gold is generally in neutral or positive oxidation states, as a result of which the position of oxygen (connecting to four zinc atoms) is not favorable.

Interesting results were observed after the geometry optimization—gold in all four positions had comparable cohesive energies (the relative difference under 3%, where all the positions except for the substitutional position of oxygen had practically the same cohesive energies), which indicated that gold is a very stable noble metal that will not interact with its vicinity in such a way as, e.g., erbium from our previous study [33]. After the geometry optimization, the models of octahedral and tetrahedral interstitial positions ended in the same structural arrangement (see Figure 2f,g), having the same cohesive and defect-formation energies—this showed that the large diameter of gold caused similar changes in the interstitial positions in the ZnO structure. The substitutional positions of zinc and oxygen in Au:ZnO had tetrahedral coordination, whereas the large interstitial voids (octahedral and tetrahedral) had a large coordination number—above 6.

Since none of the models had negative defect-formation energies, the formation of Au defects in ZnO will not be energetically favorable (or stable), meaning that the gold atoms in the ZnO structure will cause larger structural damage. Overall, the highest cohesive energies and the lowest Au defect-formation energies had the model with gold in the substitutional position of zinc (connecting to four oxygen atoms), making this position the most favorable for gold defects—the same result was observed for erbium in the previous study [33].

It is worth mentioning that the crystal ionic radius of the Au^+^ ion (151 pm [41]) is almost twice as large as that of the Zn^2+^ ion (74–88 pm [41], depending on the coordination number). The incorporation of gold thus creates strain in the crystal; therefore, some vacancies around the Au atom need to be created. The incorporation of the vacancies is also required for the charge compensation because gold is mostly present as an Au^+^ ion in the structure. For further investigation of Au:ZnO, it is hence necessary to create models with various numbers of vacancies in the vicinity of the Au atom.

### 4.2. The Theoretical Simulations of the Au Interaction with ZnO and the He^+^ Ion-Channeling Effect in Various ZnO Planes

We used two simulation programs—the stopping and range of ions into matter (SRIM) [42] and FLUX codes [32]. SRIM was used to calculate the depth-distribution profiles of Au^+^ ions in the ZnO crystal, which were compared to the experimental results of Au concentration-depth profiles. The FLUX program simulated the trajectories of high-energy He^+^ ions in various ZnO planes in channeling, or near-channeling, direction. Through the visualization of the channeling effect of He^+^ ions in pristine ZnO compared to the modification of channeling effect for damaged ZnO in various planes, we can better way interpret the influence of the differences between various used cuts of ZnO.

The implantation of Au^+^ ions with different energies mainly affects the depth of the projected range and the damage-depth profiles (depending on the portion of ion energy deposited via ionization—i.e., electronic stopping—and via collisions with the target nuclei, i.e., nuclear stopping). Different ion-implantation fluences correspond to different concentrations of Au in the buried layer as well as to the growing defects that can influence the position and structure of the implanted metal in the ZnO. Our experiments worked with three different energies of implanted ions. Using the SRIM calculation [42], we received the Au depth distribution as well as the distribution of electronic and nuclear energy stop. For all energies, 0.4 MeV, 1 MeV and 5 MeV, the values of the projected range (*R_P_*) and the standard deviation of Au ion-depth distribution (∆*R_P_*) were calculated by the SRIM code as follows: *R_P_* = 63 nm and ∆*R_P_* = 19 nm, *R_P_* = 132 nm and ∆*R_P_* = 36 nm, *R_P_*~684 nm and ∆*R_P_*~179 nm, respectively. The simulation of energy stopping shows the ratio of electronic (S_e_) and nuclear energy losses (*S_n_*)—for the energy of 0.4 MeV Au, the value of *S_e_/S_n_* is 0.16, for 1 MeV 0.28 and for 5 MeV 0.87 in the surface layer. This means that nuclear stopping prevails over electronic stopping for all selected energies. However, for the energy of 5 MeV, nuclear stopping is comparable to electronic stopping. According to the results of the SRIM simulation, it is not possible to estimate the nature of the defects, but it is possible to assume that O and Zn vacancies are created in the entire depth of penetrating Au ions, and vacancy migration is a quite complex process in ZnO, influenced by implantation parameters and more possible complex defects [43].

**Figure 3:**
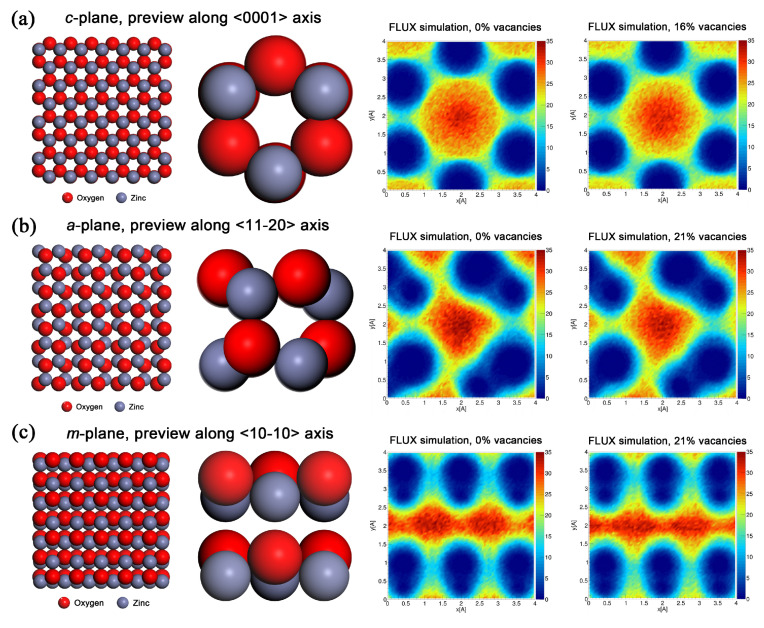
The crystal–structure models of various ZnO cuts are visualized on the left: (**a**) (0001) *c*-plane, (**b**) (11–20) *a*-plane and (**c**) (10–10) *m*-plane. The right side shows the FLUX simulation of the intensity of a 2 MeV He^+^ ion beam transmitted through a perfect ZnO crystal with various crystallographic orientations for pristine samples and for samples with introduced relative damage (measured by RBS/C) in the form of Zn vacancies. The simulation was set according to 0.4 MeV Au^+^ ion implantation with the projected range of about 63 nm (SRIM). The *x* and *y*-axis units are in Ångströms (10^−10^ m).

Transmission 2D maps of He^+^ ions through ZnO, various crystallographic orientations, were simulated using the FLUX code [32], showing the channeling effect in RBS/C analysis and the results are shown in Figure 3 (left). Here, the red color depicts the highest intensity of channeled ions in axial channels, whereas the yellow color shows decreasing transmitted ion intensity. The blue color marks completely screened areas that contain Zn atoms. Moreover, these simulations are accompanied by illustrative pictures of the ZnO structure of individual crystallographic cuts with a detail of the ZnO structure (see Figure 3 on the right). That model of the *c*-plane channel along the 〈0001〉 ZnO axis clearly shows the symmetry of the wurtzite ZnO hexagonal structure; nonpolar *a*-plane (11–20) and *m*-plane (10–10) exhibit different 2D intensity maps of channeled He^+^ ions, in good agreement with the models of crystal structures. We can follow the transmitted ion intensity decrease and narrowing of the channels in the crystalline orientations with introduced damage.

### 4.3. Experimental Results—Au:ZnO Structure Characterization by the RBS

The Au concentration-depth profiles of the as-implanted and as-annealed ZnO samples implanted with three different energies were determined by the RBS method. The resulting concentration-depth profiles and their detailed description were given in our previous papers [29,31,44]. Therefore, this paper provides a very brief overview of the findings and a comparison of the results for individual energies. The following facts have emerged from the measured experimental RBS data: (i) for two lower energies, the dopant depth profiles exhibit a shape close to the normal distribution function with the maximum concentration depth in agreement with SRIM prediction, but the Au depth profiles are broader, with slightly asymmetric tails into the depth, in comparison with the SRIM. This effect could be ascribed to the partial channeling of a small portion of implanted particles, which differs in various ZnO orientations because of the effect of the secondary channeling of the originally non-channeled particles and varying stopping powers depending on the crystallographic [29,45] orientation. (ii) For all Au concentration-depth profiles, the slight shift of the Au concentration maximum to the depth was in the depth resolution uncertainty of RBS (shown for the energies of 0.4 and 1.5 MeV in [29]). (iii) With the increasing ion-implantation fluence, the maximum is shifted closer to the surface as the structure becomes more defected, and the dopant concentration gradually increases in the buried layer during the implantation. (iv) The annealing procedure causes a slight Au depth-profile modification, which means that the Au-concentration maximum is slightly shifted towards the surface. (v) No significant differences were observed between Au concentration-depth profiles.

### 4.4. Experimental Results—Au:ZnO Structure Characterization by the RBS/Channeling

RBS/channeling spectra were measured for all the mentioned samples [29,31,44]. The values of the relative disorder were determined as the normalized yield (*χ_D_*) according to the approach presented elsewhere [44]. The normalized yields were extracted from the RBS/C spectrum as the ratio of the yield in the aligned spectrum to the yield in the random spectrum in the region of interest, which is a thin sub-surface layer. The values calculated for particular crystallographic orientations as well as for various energies are summarized in Table 3. It is clear from them that the total damage is affected by both the used fluences and energy. It is evident that the damage increases with the fluence as expected—the high fluence of 1.5 × 10^16^ ions/cm^2^ causes much greater damage than the two lower fluences. This trend does not apply to energy. It is clearly visible that 0.4 MeV Au ions damage the sub-surface layer more significantly, as the nuclear stopping is about five times higher than electronic stopping, contrary to 5 MeV Au ions, in which case the sub-surface layer is less damaged, and electronic and nuclear stopping are equal. Nuclear stopping is suggested to be mostly responsible for Zn-sublattice disordering, and it is monitored in RBS/C. This statement is supported by SRIM simulation, where the nuclear stopping value for 0.4 MeV is about 6.14 keV nm^−1^ and for 5 MeV about 3.51 keV nm^−1^ in the investigated sub-surface layer (~200 nm). For all implantation energies, the highest Zn-sublattice disorder was evidenced in *m*- and *c*-plane ZnO and the lowest for *a*-plane ZnO.

**Figure 4:**
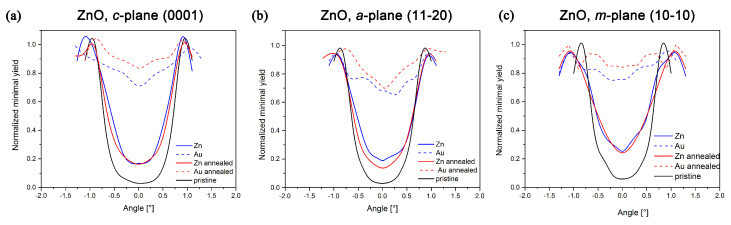
The angular scans provided in the RBS/C analysis of the as-implanted and as-annealed samples: (**a**) *c*-plane ZnO, (**b**) *a*-plane ZnO, (**c**) *m*-plane ZnO. The experimental conditions of the sample preparation: the energy of Au^+^-ion implantation was 0.4 MeV, the fluences were 5 × 10^14^ cm^−2^, and the post-implantation annealing was done at 600 °C in oxygen for 1 h.

The angular scans were performed to identify the preferential Au positioning and to provide information on the preservation of the channeling effect and the quality of Zn ordering. Figure 4 thus shows the angular scans only for the 0.4 MeV implantation energy.

Based on the results described, it is possible to clarify whether the implanted Au ions occupy Zn substitution/shadow positions or are placed in random positions in the target structure. The angular scans in RBS/C analysis were provided for 0.4 MeV Au implantation, where Au and Zn signals in the RBS spectrum are separated by kinematics and Au is positioned under the surface. In the case of 5 MeV Au-ion implantation, we studied the same energetic window in RBS/C analysis corresponding to the Zn matrix; the Au signal was not monitored as it was positioned deep in ZnO and overlapped with the Zn signal. Angular scans in 5 MeV ion-implanted ZnO thus provide information on the structural modification of the Zn-sublattice in the subsurface layer with the maximum electronic stopping. The angular scans done by RBS/C on ZnO Au 0.4 MeV implanted samples are presented in Figure 4 and it can be concluded that only a small portion of Au is placed in any preferential position; it is mostly distributed randomly, which is indicated by the fact that the normalized yield of Au in the angular scans does not copy the normalized yield of Zn. This means that only a small portion of Au is positioned in Zn-substitutional/shadowed positions. Simultaneously, this portion is even smaller with increasing Au-implantation fluence—the normalized yield of Au is enhanced within the channel (see Figure 4).

Moreover, distinct channeling effects were observed in various ZnO orientations: the mostly preserved channeling effect in *a*-plane ZnO, contrary to the narrowing and smearing of the channels in *m*- and *c*-plane ZnO. Such a phenomenon was observed in 5 MeV ion-implanted ZnO as well, depending on crystallographic orientation, but the channeling effect was deteriorated to a lesser extent due to the lower energy deposition via collisions [30]. Therefore, the angular scans for 1.5 MeV ion implantation are not presented. Since the ion fluence is high, the Au portion in interstitial positions will be even higher, and the channeling quality even lower than in the presented samples. Such results were observed in all experiments performed by Au^+^ implantations, but it is a general phenomenon, which was also evident in the Er implantation [33,46]. Looking back at Figure 3 (see the FLUX simulations—the intensity of the red color), regarding the cause of this phenomenon, we can speculate that in *a*-plane ZnO is to a greater extent enabled secondary channeling leading to lower damage in comparison to the other two ZnO planes.

### 4.5. Experimental Results—Au:ZnO Structure Characterization by TEM

**Figure 5:**
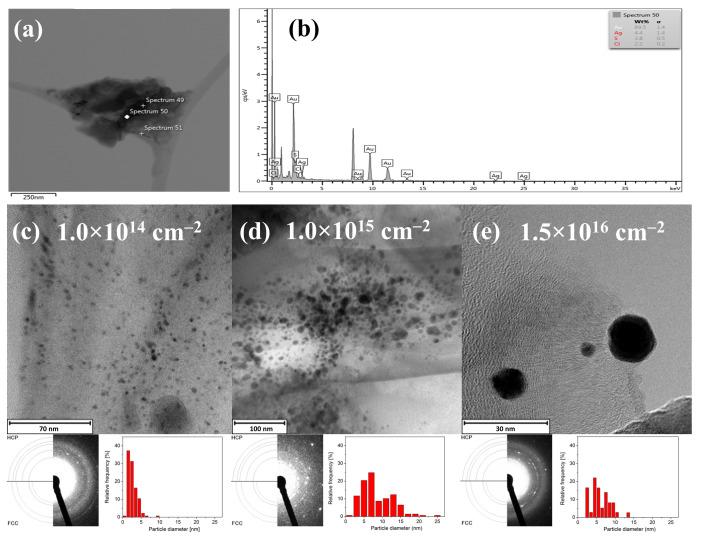
TEM images and EDS analyses of created Au nanoparticles—(**a**) Au nanoparticles (black color) placed on the Cu grid; (**b**) EDS elemental analyses at the point marked as fifty. Nanoparticles were created by ion implantation with the following conditions: the energy of 1.0 MeV, the fluence of 1.5 × 10^16^ cm^−2^, annealed at 600 °C in O_2_ atmosphere for 1 h (*m*-plane); (**c**–**e**) the shape of created Au nanoparticles—the nanoparticle size affected by implantation fluence is shown together with XRD analyses of the nanoparticles and size distribution. Nanoparticles were created by ion implantation with the following conditions: the energy of 0.4 or 1.0 MeV, fluences of 5 × 10^14^ cm^−2^, 1 × 10^15^ cm^−2^ or 1.5 × 10^16^ cm^−2^, annealed at 600 °C in an O_2_ atmosphere for 1 h. (*c*- or *m*-plane). Because in our previous work, it was confirmed that the formation of the nanoparticles is slightly affected by the planes of ZnO [44], the analyses are shown as selected ZnO crystallographic orientations.

To describe where and in which form/oxidation state the implanted Au ions are placed in the ZnO structure, TEM analyses were performed on selected samples. Since the formation of TEM lamellae by focused ion beam (FIB) using gallium ions affected the Au profiles, the powder samples etched with citric acid were used for the TEM. All crystallographic ZnO orientations implanted with Au at the energy of 1 MeV and the highest fluence of 1.5 × 10^16^ cm^−2^ were analyzed (see [44]). Using this highest fluence, the formation of gold nanoparticles was expected. In addition, two comparative samples were selected where Au was implanted with the energy of 0.4 MeV at both lower fluences, 1 × 10^14^ and 1 × 10^15^ ions/cm^2^. The results are shown in Figure 5. Figure 5a,b shows the distribution of Au on the Cu grid as well the EDS analyses performed in black particles (the spectrum 50) in Figure 5a. The EDS analyses have confirmed that the particles are metallic Au. Figure 5c–e show the steric shape of the created nanoparticles as well as the change in the size of the nanoparticles with increasing implantation fluence. The images come from HRTEM analysis; they show the interplanar distance of about 0.25–0.28 nm. They are complemented by a graph of calculated nanoparticle distribution is shown. A comparison of the depicted distributions shows that the size of spherical nanoparticles increases with increasing fluence. For the 1 × 10^14^ cm^−2^ fluence used, the size of nanoparticles is between 1 and 6 nm, while for both higher fluences may be up to 20 nm in diameter. When diffraction was performed on these noticeable Au particles, the crystal structure of gold did not correspond to the expected *fcc* arrangement but more likely to the *hcp* structure. This arrangement is a result of the surrounding wurtzite (hexagonal) structure of ZnO, in which the nanoparticles are formed.

### 4.6. Experimental Results—Au:ZnO Structure Characterization by Raman Spectroscopy

**Figure 6:**
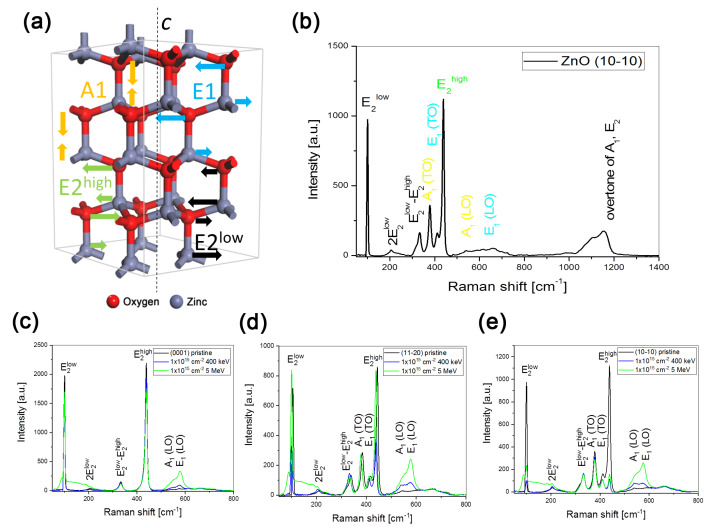
The Raman spectra of the Au:ZnO structure measured for the lower and higher applied energy of ion implantation in various cuts: (**a**) the representation of atomic displacement corresponding to active optical modes in the wurtzite ZnO structure; (**b**) all Raman vibration bands characteristic of ZnO shown in the spectrum of *m*-plane ZnO. Both figures were made according to [47]. Figures (**c**–**e**) are comparisons of the Raman spectra collected for the implantation energy of 0.4 MeV and 5.0 MeV in various ZnO cuts used. This figure includes samples implanted with the fluence of 1 × 10^15^ ions/cm^2^, with post-implantation annealing at 600 °C in an O_2_ atmosphere for 1 h.

Raman spectroscopy characterizes the vibrational dynamics of the ZnO lattice. Raman scattering is a process of inelastic scattering of incident light inducing a change in the polarizability of the bond. The scattered light consists of photons with unchanged frequency (due to Rayleigh scattering) and photons with higher and lower frequency, with the value difference from Rayleigh scattering being molecular vibration. This results in the symmetry of Raman spectra along the Rayleigh spectral line, where spectral lines with higher and lower frequency lie in the Stokes and anti-Stokes region, respectively. The final Raman spectra shown are from the Stokes region, only with the Rayleigh spectral line filtered. The wurtzite crystal structure of ZnO belongs to the C_6v_ space group with irreducible representation Γ = 2A_1_ + 2B_1_ + 2E_1_ + 2E_2_. Optically active phonon modes for the Raman spectra in the Brillouin zone include A_1_, E_1_ and double degenerate 2E_2_ (denoted as E_2_^low^ and E_2_^high^). The corresponding atomic displacements within the ZnO structure are shown in Figure 6a. A1 is the phonon mode describing vibrations of mainly oxygen atoms along the c axis, whereas E_1_ vibrations are perpendicular. As a result of these movements, oscillating polarization is induced, splitting the A_1_ and E_1_ modes into longitudinal (LO) and transversal (TO) optical modes. The E_2_ modes are nonpolar and do not result in the LO-TO splitting. The E_2_ phonon modes are characteristic of ZnO in Raman spectra, and their width and intensity are used to determine the quality of ZnO monocrystals. In the Raman spectra of ZnO bulk samples, it is possible to find six peaks with their overtones mentioned above. However, whether all of those peaks are shown depends on the measurement arrangement.

The spectral lines characterized above, as well as a visual representation of their vibrations, are depicted in Figure 6b, which shows the Raman spectrum of an *a*-plane pristine sample. The Raman spectra of the samples implanted with the lowest and highest energy using the fluence of 1 × 10^15^ cm^−^^1^ are given for different crystal planes in Figure 6c–e. Only three phonon modes, E_2_^low^, E_2_^high^ and A_1_(LO), are observed in the selected experimental arrangement, where the incident beam is focused on the *c*-plane (0001) at 100, 439 and 574 cm^−^^1^, respectively (see Figure 6c). Near 202 cm^−^^1^ and 333 cm^-1^, the overtone of the E_2_^low^ phonon mode and the multi-phonon E_2_^low^–E_2_^high^ combination are located, respectively. The wide bands in the ranges of 500–750 cm^−^^1^ and 1050–1200 cm^−^^1^ (shown only in Figure 6b) are a result of optical-acoustic and optical combinations. When the *a*-plane (11–20) or *m*-plane (10–10) of ZnO are studied with Raman spectroscopy, it is possible to observe all five phonon modes, namely A1(TO) at 380 cm^−^^1^ and E1(TO) at 410 cm^−^^1^ in addition to the three *c*-plane phonon modes (see Figure 6d,e) [48,49].

Concerning the effect of ion implantation in individual orientations, it is evident that ion implantation damages the structure of the ZnO lattice and reduces the intensity of the characteristic E|_2_^high^ phonon mode as well as the E_2_^low^ phonon mode. This effect is enhanced by an increase in the implantation fluence of Au (not shown, reported in our previous publications in agreement with other authors). When the higher and lower energies used are compared, a more significant damage, Zn disordering, is caused by the energy deposition via nuclear stopping. This assumption is confirmed by the more significant lowering of E_2_^high^ and E_2_^low^ phonons after 0.4 MeV Au-ion implantation than in the case of the 5 MeV Au ion-implanted ZnO (see Figure 6). A comparison of the E_2_^high^ intensities in all crystallographic orientations shows a decrease in the *m*-plane (10–10) orientation. Furthermore, this shows that oxygen sublattice ordering is damaged mostly only in *m*-plane. Moreover, the structural change of oxygen manifests itself in the intensity increase of A_1_(LO) and E_1_(LO) phonon modes, especially at the energy of 5 MeV. Additionally, all Raman spectra contain a wide band at 80–240 cm^−^^1^, which is attributable to a change in zinc-atom positions because of the high-energy implantation of Au ions. The Raman spectral band corresponding to the formation of the Au-O bond has not been found at any implantation energy or Au-ion fluence.

### 4.7. Experimental Results—Au:ZnO Optical Characterization by Photoluminescence spectroscopy

**Figure 7:**
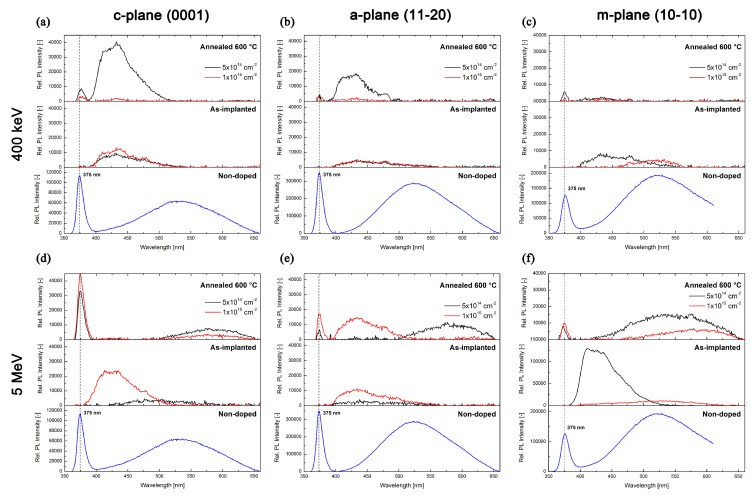
The photoluminescence spectra of undoped pristine ZnO samples and as-implanted and as-annealed ZnO samples doped with (**a**–**c**) 0.4 MeV and (**c**–**e**) 5 MeV Au^+^ ions for (**a**,**d**) *c*-plane, (**b**,**e**) *a*-plane and (**c**,**f**) *m*-plane crystallographic cuts of ZnO. The ion-implantation fluences used were 5 × 10^14^ and 1 × 10^15^ cm^−2^.

Photoluminescence spectra were measured in the UV-VIS range of ca 350–700 nm. This spectral region provides valuable information on various structural defects of ZnO. Figure 7 shows the measured luminescence spectra of the undoped pristine (blue curves) ZnO samples and as-implanted (black curves) and as-annealed (red curves) *c-, a-* and *m-plane* Au:ZnO samples doped using 5 × 10^14^ and 1 × 10^15^ cm^−^^2^ ion implantation fluences. Figure 7a–c shows the samples implanted at the energy of 0.4 MeV, and Figure 7d–f includes 5 MeV implanted samples. All the spectra were baseline-corrected to the standard sample, which makes it is possible to compare the luminescence intensities of different samples in the spectra. The measurement of photoluminescence spectra has revealed differences in the properties of the crystallographic cuts of ZnO.

The photoluminescence spectra of the pristine (non-implanted) samples showed the near-band-edge (NBE) luminescence band at around 375 nm (assigned to exciton recombination) and the broad deep-level-emission (DLE) green luminescence band at about 550 nm (attributed to transitions including oxygen-vacancy defects (V_O_) in the ZnO structure [50,51]). The intensity of the NBE luminescence bands decreased in the following order: *c*-plane > *a*-plane > *m*-plane (Figure 7a,b,d–f). This phenomenon can be explained by the strong anisotropy of light propagation in the ZnO structure, where, in some directions in the ZnO structure, the propagation of light is substantially suppressed [52]. The DLE luminescence band showed the opposite behavior, i.e., the luminescence intensity increased. Such behavior can be related to the higher concentration of oxygen defects on the nonpolar surface of the samples.

After ion implantation, the NBE (exciton-related) luminescence vanished in all crystal planes and the broad green DLE luminescence band was significantly suppressed; on the other hand, violet DLE luminescence at about 420 nm was detected, which was more intense for the higher ion-implantation fluence used (see Figure 7—the as-implanted samples). The vanishing of the 375 nm band after the ion implantation is most likely a consequence of the disturbance to the ZnO bandgap, resulting from the substantial structural damage induced by the ion implantation. The emission band at around 425 nm is assigned to the transitions, including both zinc interstitial (Zn_i_) defects and zinc vacancy (V_Zn_) defects [50,51,53].

The PL spectra have revealed that the NBE exciton-related luminescence band of Au:ZnO samples was restored for all crystal planes after the thermal annealing at 600 °C due to the recovery of the ZnO structure (see Figure 7—the as-annealed samples). After the annealing, the NBE band was restored with the highest intensity in the *c*-plane. Most of the defect-related DLE bands (425 and 520 nm) then vanished, which was most likely caused by the recovery of the structure (the disappearance of the zinc interstitials and vacancies). The disappearance of the oxygen-vacancy-related bands (520 nm) was caused by the annealing in the oxygen atmosphere. For all the crystal planes, annealing also resulted in a shift of the DLE luminescence band to around 580 nm (the yellow color), which is attributed to transition including oxygen interstitials O_i_ [10,11]. It is interesting that the exciton-related NBE luminescence band was the lowest in the *m*-plane sample. This may be caused by the substantial modification of the surface structure of *m*-plane ZnO, which was confirmed by RBS and Raman spectroscopy analyses, where the *m*-plane samples had the highest structural disorder after the ion implantation. However, further study of this phenomenon (including several possible reasons for it, such as anisotropy, crystal damage, etc.) is required.

## 5. Discussion

It follows from this experiment that gold in the form of Au(+I) ions in the ZnO structure does not enter any substitutional positions. A significant part of Au is randomly distributed, simultaneously creating clusters and nanoparticles Au(0). These observations confirm the results of a number of authors for gold in various matrices. According to [21], Au(0) and Au(+I) oxidation states are stable on Zn-vacant sites of the ZnO (0001) surface, while the higher charge states of gold are unstable. All positively charged states of gold (+I, +II and +III) can, however, be stable when replacing Zn^2+^ in bulk-terminated island sites, although higher oxidation states of Au are not energetically feasible to create. These results also partially correspond to our findings, especially with regard to the stability of the Au(0) oxidation state.

Regarding the position of Au in the ZnO structure, the experimental results were corroborated by DFT geometry optimization. The main DFT result is that the value of defect formation energy for substitutional and interstitial positions provided by the simulation is always positive and high. Therefore, we can assume that gold is distributed in the ZnO structure randomly. This assumption, based on DFT calculations, was also experimentally confirmed. Moreover, we can assume that the energy of the bandgap at any time to reduce, which is also confirmed in our experiments by luminescence, specifically the decrease in the intensity of NBE luminescence bands. According to [26], where the Au defect formation energies were calculated, the Au atoms will more likely take interstitial sites rather than Zn sites. The bandgap was found to decrease for both Au positions in ZnO. These results also correspond well with our experiments and calculations.

Since the DFT simulation was performed in the same way as the simulation of the position of the erbium ion in the ZnO structure [33], it is possible to compare the behavior and simulations of the surroundings of Er and Au ions of relatively different chemical properties. When Er^+^ ions were implanted into the ZnO structure under the same conditions, their behavior was very different. After ion implantation, they clearly preferred the zinc substitution position, and the subsequent annealing did not make them cluster and form metal bonds, but they were rather pushed out of the structure, shifting to Zn interstitial positions, depending on the annealing temperature [33,39,54]. In our opinion, the different behavior is caused by the properties of both ions, the larger mass of Au (196.7 vs. 167.3 g.mol^−^^1^ for Er), larger ionic radius of Au(+I) than Er(+III) in coordination VI (137 pm vs. 89 pm for Er^3+^ [41]) and mainly its lower affinity for oxygen (the bond dissociation energy of 223 kJ mol^−1^ for Au–O vs. 606 kJ mol^−1^ for Er–O [55]). Unlike gold, however, the erbium ion tries to form a bond with oxygen—according to the DFT simulation, it would achieve the greatest stability if surrounded by six oxygens, which is related to the preference for the octahedral arrangement of oxygen in its vicinity [33,56,57]. Since the erbium ion is highly mobile, probably thanks to the bonds to oxygen in the structure, it is strongly diffused to the surface, as confirmed by other authors [33,54]. Immobile gold ions remain at the point of impact, where they also agglomerate, forming metal bonds and nanoparticles. It is clear from the results that the simulation of the geometric vicinity is in very good agreement with the experiment. This fact can be explained by the similarity of the model and the experimental arrangement, when the ions, both during DFT simulation and ion implantation, are ‘embossed’ into the crystal structure, and the subsequent annealing works in the same way as geometry optimization, with the ion seeking the most energetically advantageous position in the structure.

Thanks to its large radius, high density and weight, low affinity for oxygen, as well as a dense network of small tetrahedral cavities in the ZnO structure, gold always forms metal particles in ZnO. This has been confirmed by many authors, albeit using various methods of Au:ZnO preparation [3,58]. The size of gold particles in ZnO varies depending on the preparation technique, and it ranges from about 1 to 100 nm [18]. Our experiment has confirmed that the used ion implantation and subsequent annealing enable the formation of nanoparticles of a smaller radius. The size of the prepared nanoparticles is related to the deposition or annealing temperature used [59]. Because of the lower annealing temperature used in our case, the Au nanoparticles formed smaller in diameter than nanoparticles reported by other authors [20]. Moreover, the presence of small Au nanoparticles dispersed in the layer due to the lower Au-ion fluences was expected because the number of nanoparticles is a growing function of ion fluence and the nanoparticle size increases mainly for higher ion-implantation currents [60]. The prepared gold nanoparticles clearly showed the relatively rare *hcp* structure for 0.4 and 1.5 MeV Au^+^ ion energy used and the fluence of ion implantation. The *hcp* arrangement is enforced by the surrounding ZnO structure during the growth of nanoparticles. Similar observations have been published for other materials such as Ge [12] and Ag [61]. In addition to the diffraction pattern, which confirms the *hcp* structure, it is possible to discuss interplanar distances, which in our case are in good agreement with the value of 0.25–0.28 nm, published in [9,10].

**Figure 8:**
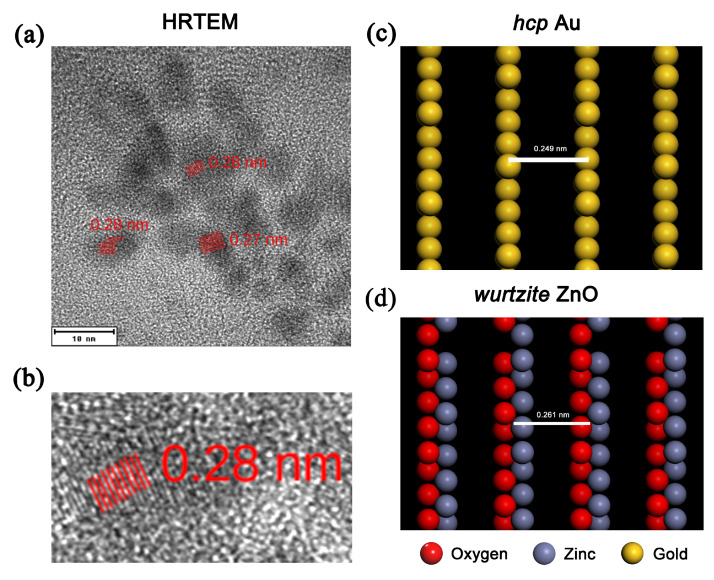
(**a**,**b**) Interplanar distances shown on HRTEM images and (**c**,**d**) model of *hcp* Au (the uncertainty of the evaluation of interplanar distances is around ±0.03 nm). The models in (**c**,**d**) were created using Accelrys Materials Studio.

However, in most of the works published so far, as well as in our work, it is not possible to determine from which direction we are looking at the particle in HRTEM analysis. For this discussion, we tried to create models of different views on interplanar distances in the *fcc* and *hcp* structures of gold. We compared these calculations in detail with the interplanar distances observed in the images (see Figure 8). The comparison showed that while the interplanar distances in the *fcc* structure resulted in low values of 0.203 and 0.235 nm (not shown), the calculations in the *hcp* structure yielded higher values of 0.222, 0.226, 0.229 and 0.249 nm, which agrees well with our measurements. ZnO in wurtzite *hcp*-type structure resulted in the values of interplanar distances of 0.158, 0.261 and 0.282 nm. This also points out to the fact that the structure of prepared gold nanoparticles embedded in wurtzite-type ZnO may have an *hcp* structural arrangement.

The question is whether this unusual structure of gold could provide new applications, for example, in biomedicine, as is the case with the mentioned materials.

It is clear from the experiments performed that gold implantation into the ZnO structure causes the formation of structural defects and a change in the properties of ZnO. Hence far, the majority of published works deal only with the standard ZnO *c*-plane orientation. In addition, we will discuss the changes of the optical properties (that corresponds to the internal atomic and electronic structure) and structural changes caused by the implantation of gold into the differently oriented planes of the ZnO. If we focus on the differences in the properties of the luminescence spectra of individual cuts, it has been confirmed that NBE luminescence at 375 nm is lowered after the ion implantation because of the presence of defects and then partially recovered by the annealing process. This effect is the same in all ZnO crystallographic cuts. This phenomenon is generally known and is related to the reduction of the energy, prohibited due to the disruption of the structure by the impact of the implanted particle. If this effect is compared for different selected energies of ion implantation, the damage is lower when higher energy is used. This effect is a manifestation of greater damage to the surface layer at lower energy used, i.e., the reduction in luminescence due to absorption—the scattering of the damaged surface.

DLE luminescence, with two main bands at 425 and 520 nm, is much more influenced by the selected crystallographic cuts. The 425 nm band, associated with Zn at interstitial positions or with Zn vacancies, is prominent in all cuts after ion implantation. Subsequent annealing in oxygen causes changes in the band around 500 nm, which is related to oxygen in the interstitial positions. The changes are the most pronounced in the *m*-plane. These results are in agreement with RBS/C, showing higher structural disorder. Nevertheless, this means that the higher structural disorder, contributing to enhanced yield in the aligned spectrum (the extended defect), causing the stress and deteriorating channeling effect (which can be seen in the *m*-plane ZnO as well as with the Raman spectra), has exhibited a significant modification in the *m*-plane ZnO, corresponding to the creation of oxygen vacancies and clusters in this nonpolar surface.

## 6. Conclusions

This study was focused on the investigation of the position and structural arrangement of gold implanted with different energies and fluences into the wurtzitic structure of ZnO. The study was performed both theoretically and experimentally. The theoretical DFT simulations, i.e., mostly the calculations of the defect-formation energies connected with the incorporation of gold into various positions in the wurtzite ZnO structure, have shown that gold atoms do not have any preferential position in the ZnO structure and do not even bind with the oxygen atoms in a certain structural coordination.

In accordance with theoretical calculations, experimental results from TEM and HRTEM have shown that gold is placed in the structure of ZnO in the form of small metal clusters, whose sizes increase with increasing ion-implantation fluence—determined in 0.4 MeV Au-implanted samples. For the low ion-implantation fluence of 5 × 10^14^ ions/cm^2^, the size of the GNPs was half smaller than for the 1.5 × 10^16^ ions/cm^2^. All gold nanoparticles exhibited the unusual *hcp* structure, which was caused by the reluctance of gold to place itself in the interstitial or substitutional positions of ZnO and to form bonds with oxygen. In addition, since the gold atoms were implanted in a different arrangement of the crystal surface of the single ZnO crystal relative to the ion beam, it was shown that the impact of ions on the structure causes various defects. Higher damage formation, demonstrated jointly by RBS/C techniques, Raman spectroscopy and photoluminescence, was observed in *m*-plane, in contrast to *c*- and *a*-plane ZnO crystallographic orientations.

In the structure of ZnO, thin films containing nanoparticles of unusual, hexagonal gold was created by ion implantation. In the selected ZnO cuts, it was possible to influence the number of oxygen defects by gold ion implantation and thus change the luminescence in the VIS. Gold nanoparticles embedded in ZnO are known to have a practical use in advanced photonics applications such as photocatalysts, sensors and photovoltaic devices, mainly thanks to the surface-plasmon-resonance properties of gold. Altering the structure of gold nanoparticles embedded in semiconductors changes the resulting properties of such systems and results in new uses for gold nanoparticles.

## Figures and Tables

**Table 1:** A description of the fractional coordinates of atoms in the ZnO model [35] together with four different Au positions in ZnO.

Unit Cell	Atom	Positions in Fractional Coordinates (*x, y, z*) for Hexagonal-Cell Representation *
Single-cell	Zn	(0.33333333, 0.66666667, 0.00000000)
	O	(0.33333333, 0.66666667, 0.38230000)
Supercell 2 × 2 × 2	Au in Zn position	(0.33333333, 0.66666667, 0.75000000)
	Au in octahedral void	(0.50000000, 0.50000000, 0.56550000)
	Au in tetrahedral void	(0.33333333, 0.66666667, 0.61926400)
	Au in O position	(0.33333333, 0.66666667, 0.44050000)

* The precision of 8 decimal places is the same as that used in the simulations.

**Table 2:** The calculated cohesive energies (E_Coh_) of geometry-optimized ZnO and Au:ZnO supercell models, together with the calculated gold defect-formation energies.

	Without Geometry Optimization		With Geometry Optimization	
Structure	Calculated Cohesive Energy (eV/atom) *	Au Defect Formation Energy (eV)	Calculated Cohesive Energy (eV/atom) *	Au Defect Formation Energy (eV)
ZnO (exp.) [40]	3.76	-	3.76	-
ZnO (calc.)	3.53 **	-	3.53 **	-
Au-Zn: ZnO	3.26	+7.0	3.33	+4.9
Au-OctVoid: ZnO	3.17	+11.2	3.33	+5.9
Au-TetrVoid: ZnO	2.52	+32.5	3.33	+5.9
Au-O: ZnO	2.97	+15.3	3.24	+6.4

* In the case of the calculated cohesive energies of Au-doped ZnO, only the energy in the form of eV/atom is considered because the formal unit is no longer ZnO. ** The difference in the values of the calculated cohesive energies of undoped ZnO before and after geometry optimization is negligible—in the order of 10^−6^ eV/atom.

**Table 3:** The minimum yield (χ_D_) is presented for as-implanted and as-annealed samples for all crystallographic orientations of ZnO samples. The values were obtained from RBS/C analysis after Au-ion implantation with various energies and fluences [29,31,38].

Ion Energy/Fluence MeV/(ions/cm^2^)	*χ_D_ - c*-plane (0001)		*χ_D_ - a*-plane (11–20)		*χ_D_ - m*-plane (10–10)	
	Implanted	Annealed	Implanted	Annealed	Implanted	Annealed
	(%)	(%)	(%)	(%)	(%)	(%)
Pristine	3 ± 0.5	–	3 ± 0.5	–	4 ± 0.5	–
Au 0.4 MeV/5 × 10^14^	16 ± 0.3	15 ± 0.3	13 ± 0.3	11 ± 0.2	22 ± 0.4	22 ± 0.4
Au 5 MeV/5 × 10^14^	9 ± 0.5	7 ± 0.5	5 ± 0.5	4 ± 0.5	15 ± 1	10 ± 1
Au 0.4 MeV/1 × 10^15^	32 ± 0.6	27 ± 0.5	21 ± 0.4	19 ± 0.4	36 ± 0.7	30 ± 0.6
Au 5 MeV/1 × 10^15^	10 ± 1	8 ± 0.5	5 ± 0.5	4 ± 0.5	17 ± 1	16 ± 1
Au 1 MeV/1.5 × 10^16^	72 ± 4.0	55 ± 2.0	51 ± 2.0	45 ± 2.0	65 ± 2.0	62 ± 2.0

## References

[1] Dykman L.A., Khlebtsov N.G. (2019). Gold nanoparticles in chemo-, immuno-, and combined therapy: Review. Biomed. Opt. Express.

[2] Sztandera K., Gorzkiewicz M., Klajnert-Maculewicz B. (2019). Gold Nanoparticles in Cancer Treatment. Mol. Pharm..

[3] Daruich De Souza C., Ribeiro Nogueira B., Rostelato M.E.C.M. (2019). Review of the methodologies used in the synthesis gold nanoparticles by chemical reduction. J. Alloy. Compd..

[4] Yang X., Yang M., Pang B., Vara M., Xia Y. (2015). Gold Nanomaterials at Work in Biomedicine. Chem. Rev..

[5] Adams M. (2015). Modern Nanoparticles Technology.

[6] Hu M., Chen J., Li Z.-Y., Au L., Hartland G.V., Li X., Marquez M., Xia Y. (2006). Gold nanostructures: Engineering their plasmonic properties for biomedical applications. Chem. Soc. Rev..

[7] Narayanan R., El-Sayed M.A. (2005). Catalysis with Transition Metal Nanoparticles in Colloidal Solution: Nanoparticle Shape Dependence and Stability. J. Phys. Chem. B.

[8] Kumar G., Tibbitts L., Newell J., Panthi B., Mukhopadhyay A., Rioux R.M., Pursell C.J., Janik M., Chandler B.D. (2018). Evaluating differences in the active-site electronics of supported Au nanoparticle catalysts using Hammett and DFT studies. Nat. Chem..

[9] Huang X., Li S., Huang Y., Wu S., Zhou X., Li S., Gan C.L., Boey F., Mirkin C.A., Zhang H. (2011). Synthesis of hexagonal close-packed gold nanostructures. Nat. Commun..

[10] Jany B.R., Gauquelin N., Willhammar T., Nikiel M., van den Bos K.H.W., Janas A., Szajna K., Verbeeck J., Van Aert S., Van Tendeloo G. (2017). Controlled growth of hexagonal gold nanostructures during thermally induced self-assembling on Ge(001) surface. Sci. Rep..

[11] Marshall A.F., Goldthorpe I.A., Adhikari H., Koto M., Wang Y.-C., Fu L., Olsson E., McIntyre P.C. (2010). Hexagonal Close-Packed Structure of Au Nanocatalysts Solidified after Ge Nanowire Vapor−Liquid−Solid Growth. Nano Lett..

[12] Marshall A.F., Thombare S.V., McIntyre P.C. (2015). Crystallization Pathway for Metastable Hexagonal Close-Packed Gold in Germanium Nanowire Catalysts. Cryst. Growth Des..

[13] Hartland G.V. (2011). Optical Studies of Dynamics in Noble Metal Nanostructures. Chem. Rev..

[14] Chen F., Amekura H., Jia Y. (2020). Overview of Ion Beam Produced Dielectric Waveguides. Ion Irradiation of Dielectrics for Photonic Applications.

[15] Lorenz K., Peres M., Franco N., Marques J.G., Miranda S.M.C., Magalhães S., Monteiro T., Wesch W., Alves E., Wendler E., Teherani F.H., Look D.C., Rogers D.J. Radiation damage formation and annealing in GaN and ZnO. Proceedings of the SPIE OPTO.

[16] Agulló-López F. (1995). Insulating Materials for Optoelectronics: New Developments.

[17] Kucheyev S.O., Jagadish C. (2006). Ion Implantation into ZnO. Zinc Oxide Bulk, Thin Films and Nanostructures.

[18] Stepanov A.L., Khaibullin R.I., Can N., Ganeev R.A., Ryasnyansky A.I., Buchal C., Uysal S. (2004). Application of ion implantation for synthesis of copper nanoparticles in a zinc oxide matrix for obtaining new nonlinear optical materials. Tech. Phys. Lett..

[19] Chamorro W., Ghanbaja J., Battie Y., Naciri A.E., Soldera F., Mücklich F., Horwat D. (2016). Local Structure-Driven Localized Surface Plasmon Absorption and Enhanced Photoluminescence in ZnO-Au Thin Films. J. Phys. Chem. C.

[20] Pereira-Silva P., Borges J., Rodrigues M.S., Oliveira J.C., Alves E., Barradas N.P., Dias J.P., Cavaleiro A., Vaz F. (2020). Nanocomposite Au-ZnO thin films: Influence of gold concentration and thermal annealing on the microstructure and plasmonic response. Surf. Coat. Technol..

[21] Phala N.S., Klatt G., van Steen E., French S.A., Sokol A.A., Catlow C.R.A. (2005). The nature of the oxidation states of gold on ZnO. Phys. Chem. Chem. Phys..

[22] Li Z., Li Y., Li J. (2012). Support effects on the dissociation of hydrogen over gold clusters on ZnO(101) surface: Theoretical insights. J. Chem. Phys..

[23] Méndez-Reyes J.M., Monroy B.M., Bizarro M., Güell F., Martínez A., Ramos E. (2015). Gold as an intruder in ZnO nanowires. Phys. Chem. Chem. Phys..

[24] Liu M.-H., Chen Y.-W., Liu X., Kuo J.-L., Chu M.-W., Mou C.-Y. (2016). Defect-Mediated Gold Substitution Doping in ZnO Mesocrystals and Catalysis in CO Oxidation. ACS Catal..

[25] Liu M.-H., Chen Y.-W., Lin T.-S., Mou C.-Y. (2018). Defective Mesocrystal ZnO-Supported Gold Catalysts: Facilitating CO Oxidation via Vacancy Defects in ZnO. ACS Catal..

[26] Li G., Ahmoum H., Liu S., Liu S., Su’ait M.S., Boughrara M., Kerouad M., Wang Q. (2019). Theoretical insight into magnetic and thermoelectric properties of Au doped ZnO compounds using density functional theory. Phys. B Condens. Matter.

[27] Goyenola C., Stafström S., Hultman L., Gueorguiev G.K. (2012). Structural Patterns Arising during Synthetic Growth of Fullerene-Like Sulfocarbide. J. Phys. Chem. C.

[28] Gueorguiev G.K., Czigány Z.S., Furlan A., Stafström S., Hultman L. (2011). Intercalation of P atoms in Fullerene-like CPx. Chem. Phys. Lett..

[29] Macková A., Malinskỳ P., Jagerová A., Mikšová R., Nekvindová P., Cajzl J., Böttger R., Akhmadaliev S. (2019). Au incorporation into various ZnO crystallographic cuts realised by ion implantation–ZnO damage characterization. Vacuum.

[30] Jagerová A., Malinský P., Mikšová R., Nekvindová P., Cajzl J., Ryšánek P., Macková A. (2020). High energy Au+ ion implantation of polar and nonpolar ZnO—Structure modification and optical properties. Surf. Interface Anal..

[31] Jagerová A., Malinskỳ P., Cutroneo M., Nekvindová P., Cajzl J., Michalcová A., Macková A. (2020). Non-polar ZnO facet implanted with Au ions and subsequently modified using energetic O ion irradiation. Nucl. Instrum. Methods Phys. Res. Sect. B Beam Interact. Mater. At..

[32] Smulders P.J.M., Boerma D.O. (1987). Computer simulation of channeling in single crystals. Nucl. Instrum. Methods Phys. Res. Sect. B Beam Interact. Mater. At..

[33] Nekvindová P., Cajzl J., Macková A., Malinský P., Oswald J., Böttger R., Yatskiv R. (2020). Er implantation into various cuts of ZnO—Experimental study and DFT modelling. J. Alloy. Compd..

[34] Clark S.J., Segall M.D., Pickard C.J., Hasnip P.J., Probert M.I.J., Refson K., Payne M.C. (2005). First principles methods using CASTEP. Z. Krist. Cryst. Mater..

[35] Sowa H., Ahsbahs H. (2006). High-pressure X-ray investigation of zincite ZnO single crystals using diamond anvils with an improved shape. J. Appl. Crystallogr..

[36] Perdew J.P. (1991). Unified Theory of Exchange and Correlation Beyond the Local Density Approximation In Electronic Structure of Solids. Electron. Struct. Solids.

[37] Nastasi M.A., Mayer J.W., Wang Y. (2015). Ion Beam Analysis: Fundamentals and Applications.

[38] Jagerová A., Malinskỳ P., Mikšová R., Nekvindová P., Cajzl J., Akhmadaliev S., Holỳ V., Macková A. (2019). Distinct defect appearance in Gd implanted polar and nonpolar ZnO surfaces in connection to ion channeling effect. J. Vac. Sci. Technol. A Vac. Surf. Film.

[39] Azarov A.Y., Hallén A., Du X.L., Rauwel P., Kuznetsov A.Y., Svensson B.G. (2014). Effect of implanted species on thermal evolution of ion-induced defects in ZnO. J. Appl. Phys..

[40] Jaffe J.E., Snyder J.A., Lin Z., Hess A.C. (2000). LDA and GGA calculations for high-pressure phase transitions in ZnO and MgO. Phys. Rev. B.

[41] Shannon R.D. (1976). Revised effective ionic radii and systematic studies of interatomic distances in halides and chalcogenides. Acta Crystallogr. Sect. A.

[42] Ziegler J.F., Ziegler M.D., Biersack J.P. (2010). SRIM—The stopping and range of ions in matter (2010). Nucl. Instrum. Methods Phys. Res. Sect. B Beam Interact. Mater. At..

[43] Azarov A.Y., Wendler E., Kuznetsov A.Y., Svensson B.G. (2014). Crucial role of implanted atoms on dynamic defect annealing in ZnO. Appl. Phys. Lett..

[44] Mackova A., Jagerová A., Malinsky P., Cutroneo M., Flaks J., Nekvindova P., Michalcova A., Holý V., Košutová T. (2020). Nanostructures in various Au ion-implanted ZnO facets modified using energetic O ions. Phys. Chem. Chem. Phys..

[45] Nordlund K., Djurabekova F., Hobler G. (2016). Large fraction of crystal directions leads to ion channeling. Phys. Rev. B.

[46] Macková A., Malinský P., Jagerová A., Mikšová R., Nekvindová P., Cajzl J., Rinkevičiūtė E., Akhmadaliev S. (2019). Damage formation and Er structural incorporation in m-plane and a-plane ZnO. Nucl. Instrum. Methods Phys. Res. Sect. B Beam Interact. Mater. At..

[47] Schumm M. (2008). ZnO-based Semiconductors Studied by Raman Spectroscopy: Semimagnetic Alloying, Doping, and Nanostructures.

[48] Kennedy J., Sundrakannan B., Katiyar R.S., Markwitz A., Li Z., Gao W. (2008). Raman scattering investigation of hydrogen and nitrogen ion implanted ZnO thin films. Curr. Appl. Phys..

[49] Alim K.A., Fonoberov V.A., Shamsa M., Balandin A.A. (2005). Micro-Raman investigation of optical phonons in ZnO nanocrystals. J. Appl. Phys..

[50] Ratajczak R., Guziewicz E., Prucnal S., Łuka G., Böttger R., Heller R., Mieszczynski C., Wozniak W., Turos A. (2018). Luminescence in the Visible Region from Annealed Thin ALD-ZnO Films Implanted with Different Rare Earth Ions. Phys. Status Solidi (A).

[51] Alvi N.H., ul Hasan K., Nur O., Willander M. (2011). The origin of the red emission in n-ZnO nanotubes/p-GaN white light emitting diodes. Nanoscale Res. Lett.

[52] Wang H.H., Tian J.S., Chen C.Y., Huang H.H., Yeh Y.C., Deng P.Y., Chang L., Chu Y.H., Wu Y.R., He J.H. (2015). The Effect of Tensile Strain on Optical Anisotropy and Exciton of m-Plane ZnO. IEEE Photonics J..

[53] Ahn C.H., Kim Y.Y., Kim D.C., Mohanta S.K., Cho H.K. (2009). A comparative analysis of deep level emission in ZnO layers deposited by various methods. J. Appl. Phys..

[54] Azarov A., Galeckas A., Hallén A., Kuznetsov A., Monakhov E., Svensson B.G. (2015). Optical activity and defect/dopant evolution in ZnO implanted with Er. J. Appl. Phys..

[55] Rumble J.R., Lide D.R., Bruno T.J. (2018). CRC Handbook of Chemistry and Physics: A Ready-Reference Book of Chemical and Physical Data.

[56] Alkahtani E.A., Merad A.E., Boufatah M.R., Benosman A. (2017). DFT investigation of structural, electronic and optical properties of pure and Er-doped ZnO: Modified Becke-Johnson exchange potential. Optik.

[57] Jalilian J., Fakhri S., Zolfaghari A. (2018). Comment on “DFT investigation of structural, electronic and optical properties of pure and Er-doped ZnO: Modified Becke-Johnson exchange potential”. Optik.

[58] Khan T., Ullah N., Khan M.A., Mashwani Z.-R., Nadhman A. (2019). Plant-based gold nanoparticles; a comprehensive review of the decade-long research on synthesis, mechanistic aspects and diverse applications. Adv. Colloid Interface Sci..

[59] Zhang X.D., Wu P., Shen Y.Y., Zhang L.H., Xue Y.H., Zhu F., Zhang D.C., Liu C.L. (2011). Structural and optical properties of Au-implanted ZnO films. Appl. Surface Sci..

[60] Popok V.N., Kumar V., Chaudhary B., Sharma V., Verma K. (2019). High-Fluence Ion Implantation of Polymers: Evolution of Structure and Composition. Radiation Effects in Polymeric Materials.

[61] Chakraborty I., Carvalho D., Shirodkar S.N., Lahiri S., Bhattacharyya S., Banerjee R., Waghmare U., Ayyub P. (2011). Novel hexagonal polytypes of silver: Growth, characterization and first-principles calculations. J. Phys. Condens. Matter.

